# Immune marker expression of irradiated mesothelioma cell lines

**DOI:** 10.3389/fonc.2022.1020493

**Published:** 2022-10-31

**Authors:** Faith Chang, Synat Keam, Tracy Seymour Hoang, Jenette Creaney, Suki Gill, Anna K. Nowak, Martin Ebert, Alistair M. Cook

**Affiliations:** ^1^ National Centre for Asbestos Related Diseases, Institute for Respiratory Health, Perth, WA, Australia; ^2^ School of Biomedical Sciences, University of Western Australia, Perth, WA, Australia; ^3^ Medical School, University of Western Australia, Perth, WA, Australia; ^4^ School of Physics, Mathematics and Computing, University of Western Australia, Perth, WA, Australia; ^5^ Department of Radiation Oncology, Sir Charles Gairdner Hospital, Perth, WA, Australia; ^6^ Department of Medical Oncology, Sir Charles Gairdner Hospital, Perth, WA, Australia

**Keywords:** radiotherapy, immune checkpoint inhibition, MHC, PD-L1, mesothelioma

## Abstract

**Background:**

Though immune checkpoint inhibition has recently shown encouraging clinical efficacy in mesothelioma, most patients do not respond. Combining immune checkpoint inhibition with radiotherapy presents an attractive option for improving treatment responses owing to the various immunomodulatory effects of radiation on tumors. However, the ideal dosing and scheduling of combined treatment remains elusive, as it is poorly studied in mesothelioma. The present study characterizes the dose- and time-dependent changes to expression of various immune markers and cytokines important to antitumor responses following irradiation of mesothelioma cell lines.

**Methods:**

Two murine (AB1, AE17) and two human (BYE, JU77) mesothelioma cell lines were treated with titrated gamma-radiation doses (1-8 Gy) and the expression of MHC class-I, MHC class-II and PD-L1 was measured over a series of post-irradiation timepoints (1-72 hours) by flow cytometry. Levels of cytokines IL-1α, IL-1β, IL-6, IL-10, IL-12p70, IL-17A, IL-23, IL-27, MCP-1, IFN-β, IFN-γ, TNF-α, and GM-CSF were measured by multiplex immunoassay in murine cell lines following 8 Gy radiation.

**Results:**

Following irradiation, a dose-dependent upregulation of MHC-I and PD-L1 was observed on three of the four cell lines studied to varying extents. For all cell lines, the increase in marker expression was most pronounced 72 hours after radiation. At this timepoint, increases in levels of cytokines IFN-β, MCP-1 and IL-6 were observed following irradiation with 8 Gy in AB1 but not AE17, reflecting patterns in marker expression.

**Conclusions:**

Overall, this study establishes the dose- and time-dependent changes in immune marker expression of commonly studied mesothelioma cell lines following radiation and will inform future study into optimal dosing and scheduling of combined radiotherapy and immune checkpoint inhibition for mesothelioma.

## Introduction

Mesothelioma is an aggressive, incurable cancer in urgent need of more effective therapies. Mesothelioma may develop in any mesothelial surface of the body, but most commonly it arises in the lung pleura following inhalation of asbestos, which acts as a carcinogen (1). The long average latency of 40 years, as well as insidious and non-specific onset of symptoms of this disease, mean that diagnosis usually occurs at an advanced stage where tumor removal is impossible, and treatment options are limited (1). For this reason, mesothelioma remains one of the deadliest cancers, with an untreated median overall survival (mOS) of nine months (2). For several decades, treatment has largely been limited to cytotoxic chemotherapy with cisplatin and pemetrexed, which only slightly improves mOS to twelve months (2). However, immune checkpoint inhibition (ICI) has recently shown encouraging clinical benefit in mesothelioma (3).

Immune checkpoints such as cytotoxic T-lymphocyte-associated protein-4 (CTLA-4), and the programmed death protein-1 (PD-1)/programmed death ligand-1 (PD-L1) signaling axis are co-inhibitory pathways that physiologically prevent inappropriate activation of immune responses, but are often upregulated by cancers to prevent or dampen T-cell activation and thus suppress antitumor activity (4). Inhibition of these checkpoints by ICI can restore or enhance tumor immunogenicity and T-cell activity against cancer (4). The recent phase 3 Checkmate 743 trial found that combined PD-1 and CTLA-4 blockade meaningfully improved survival rates over pemetrexed and platinum chemotherapy in the first-line setting for malignant pleural mesothelioma – leading to approval of this treatment strategy for clinical use (3). However, successful treatment occurred predominantly in the rarer, non-epithelioid mesothelioma subtypes that are often refractory to chemotherapy. The majority of patients either do not respond or acquire resistance to treatment, highlighting the need to find strategies to sensitize a greater proportion of patients to ICI.

The administration of other treatment modalities in conjunction with ICI, such as radiotherapy (RT), may increase both the rate and durability of responses. In mesothelioma, RT is used routinely during surgical resection as well as palliatively to assist in symptoms of pain and dyspnoea (5). Though conventionally exploited for its ability to effect DNA damage and cell death (6), recently RT has been found to produce a host of immunomodulatory effects on tumors through altering the tumor microenvironment (7, 8), initiating immunogenic cell death (9, 10), and altering the surface expression of immune markers, such as major histocompatibility complex (MHC) molecules and PD-L1, on cancer cells (11–14). These effects, as well as the relatively limited systemic toxicities of RT compared to other treatments such as chemotherapy, provide strong rationale for investigating how RT might boost ICI responses (15).

Preclinical studies in some cancers have shown improved efficacy of combined RT and ICI over either treatment alone (16–20), and combined RT and ICI is gradually being translated into the clinical setting (21). At present, however, preclinical studies combining RT and ICI in mesothelioma are limited. In one study using the AB12 murine mesothelioma model, hypo-fractionated RT (5 Gy x 3) followed by anti-CTLA-4 antibody led to abscopal effects, increased T cell infiltration, and increases in immune-related gene expression and cytokine production (22). Another study assessing a similar schedule of 5 Gy x 3 followed by anti-CTLA-4 in the AE17 model of mesothelioma found significantly smaller tumors following combined treatment compared to either treatment alone (23). Though such results are encouraging, more work is needed to assess a larger range of doses and sequences, to gain a more comprehensive understanding of the mechanisms by which combined RT and ICI can augment antitumor immune responses. To our knowledge, the immunomodulatory effects of radiation on mesothelioma cells over time and in the context of carefully titrated doses have not previously been investigated. Such information is essential to inform the design of future preclinical and clinical studies in mesothelioma. The present study therefore aimed to measure the changes to the surface expression of several immune markers relevant to ICI; namely, MHC class-I (MHC-I), MHC class-II (MHC-II) and PD-L1, following irradiation of both murine and human mesothelioma cell lines. In addition, we aimed to characterize the cytokine profile of irradiated cell lines to find potential mechanisms underlying changes to surface expression.

## Materials and methods

### Cell lines and culture

Murine mesothelioma cell lines AB1 and AE17 were generated as previously described (24). Human mesothelioma cell lines BYE10412 (BYE) and JU77, generated from patient malignant pleural effusions using methods described by Manning et al. (25), were obtained from the National Centre for Asbestos Related Disease (NCARD) biobank. Details of cell lines are summarized in **Supplementary Table 1**. Cells were cultured in R10, consisting of RPMI 1640 medium (Gibco, Thermo Fisher, MA, USA) supplemented with 20 mM N-2-hydroxyethylpiperazine-N’-2-ethanesulfonic acid (HEPES) (Gibco), 100 U/mL benzylpenicillin, 50 mg/mL gentamicin, 0.05 mM 2-mercaptoethanol (2-ME), and 10% neonatal calf serum (NCS). Cells were maintained in a humidified atmosphere at 37 °C with 5% CO2 and media was replaced twice a week.

### IFN-γ stimulation

Murine mesothelioma cell lines were treated with 10 ng/mL recombinant mouse interferon-gamma (IFN-γ) (Cat#200-16, Shenandoah Biotechnology, PA, USA) and incubated for 48 h to induce MHC-I/II and PD-L1 expression. Similarly, human mesothelioma cell lines were subject to a 48 h stimulation with 100 ng/mL recombinant human IFN-γ (Cat#PHC4031, Gibco, Thermo Fisher).

### Irradiation

At 80% confluency, cells in culture were harvested and resuspended in R10 at 1 × 10^6^ cells/mL. Cells were irradiated in separate 50 mL tubes by a caesium-137 source at a dose rate of 3.78 Gy/min (Gammacell 3000 SN #0211, Nordion, Ottawa, Canada) at room temperature with doses of 1, 2, 4, 6, and 8 Gy. As the actual radiation dose administered may vary by ± 15% depending on position in the rotating irradiation cannister, tubes were positioned centrally for all irradiations. Immediately after radiation 1 × 10^6^ cells were seeded into separate T175 culture flasks for each timepoint. Cells were then incubated for 1, 6, 24, 48 or 72 h post-irradiation. At the completion of each timepoint, 0.5 × 10^6^ cells were collected per dose and cryopreserved at -80°C until analysis. For cytokine studies, cells were irradiated at 8 Gy and incubated for 72 h, after which cell culture supernatant was collected, centrifuged (300 RCF, three minutes) to remove debris, and stored at -80°C until analysis. All irradiations were conducted in triplicate.

### Flow cytometry

Surface expression of MHC-I, MHC-II and PD-L1 was assessed on irradiated cell lines by flow cytometry. Frozen samples were thawed in a 37°C water bath for one minute, then transferred to fresh 15 mL tubes. Cells were washed in 10 mL R10 by centrifugation (300 RCF, three minutes, max brake). Each sample was resuspended in 200 µl R10, transferred to a 96 well plate, and washed by centrifugation (300 RCF, three minutes, max brake). Samples were washed twice with 200 µL/well PBS (300 RCF, three minutes, max brake). Fixable Viability Dye eFluorTM 506 (eF506) diluted 1/1600 in 20 µL PBS was added to the appropriate wells, and samples were incubated for 20 minutes in the dark at room temperature. Samples were then washed twice in 200 µL flow buffer (PBS/2% NCS/5mM EDTA)/well. Samples were stained with 20 µL of appropriate antibody cocktail (**Supplementary Table 1**) and incubated for a minimum of 30 minutes at room temperature in the dark. Following staining, samples were washed twice (300 RCF, 3 minutes, max brake) with 200 µL/well flow buffer, then resuspended in 50 µL/well 1X BD Stabilizing Fixative diluted in 5mM EDTA before analysis.

Compensation was performed using singly stained UltraComp eBeads (eBioscience, San Diego, CA). Gating was optimized using FMO controls for each marker (gating strategies are presented in **Supplementary Figure 2**). Where appropriate, IFNγ-stimulated mesothelioma cells (AB1, AE17 and JU77) were used as biological positive controls for each experiment. As MHC-II was not expressed on either murine cell line following IFN-γ, mouse splenocytes were selected as a technical positive staining control for MHC-II antibody.

Samples were acquired using the FACSCantoII (BD Biosciences, NJ, USA) and FACSDiva software (BD Biosciences), and a minimum of 10,000 live events were recorded per sample. Data were analyzed using FlowJo software, version 10.8.0 (Treestar, OR, USA) to generate values for the percentage of cells positive for MHC-I, MHC-II and PD-L1 expression and median fluorescence intensity (MFI). Normalized MFI (nef) was generated by dividing the MFI of each sample with MFI of an unstained control. All radiation doses and timepoints within any individual irradiation experimental repeat were stained and data acquired in the same session.

### Multiplex immunoassay

Levels of inflammatory cytokines (IL-1α, IL-1β, IL-6, IL-10, IL-12p70, IL-17A, IL-23, IL-27, MCP-1, IFN-β, IFN-γ, TNF-α, and GM-CSF) were measured in murine mesothelioma cell lines using the LEGENDplex™ 13-plex Mouse Inflammation Panel with Filter Plate (Cat#740150, Biolegend, CA, USA). The assay was performed according to manufacturer’s instructions. Standards and samples were assayed in duplicate. Samples were acquired with the FACSCantoII and FACSDiva software (BD Biosciences). Standard curves and cytokine data were generated using the free cloud-based LEGENDplex™ Data Analysis Software Suite (Biolegend).

### Statistical analysis

Data were analyzed and visualized using GraphPad Prism software (version 9.1.2). Immune marker expression was analyzed by two-way analysis of variance (ANOVA), followed by multiple pairwise comparisons with Tukey adjustment. The non-parametric Mann-Whitney U test was used to compare concentrations of a given cytokine in un-irradiated (0 Gy) versus irradiated (8 Gy) samples. Results are presented as mean ± one standard deviation of *n = 3* independent experimental repeats in all figures. P < 0.05 was considered statistically significant.

## Results

### Basal and IFNγ-induced expression of immune markers

**Figure 1:**
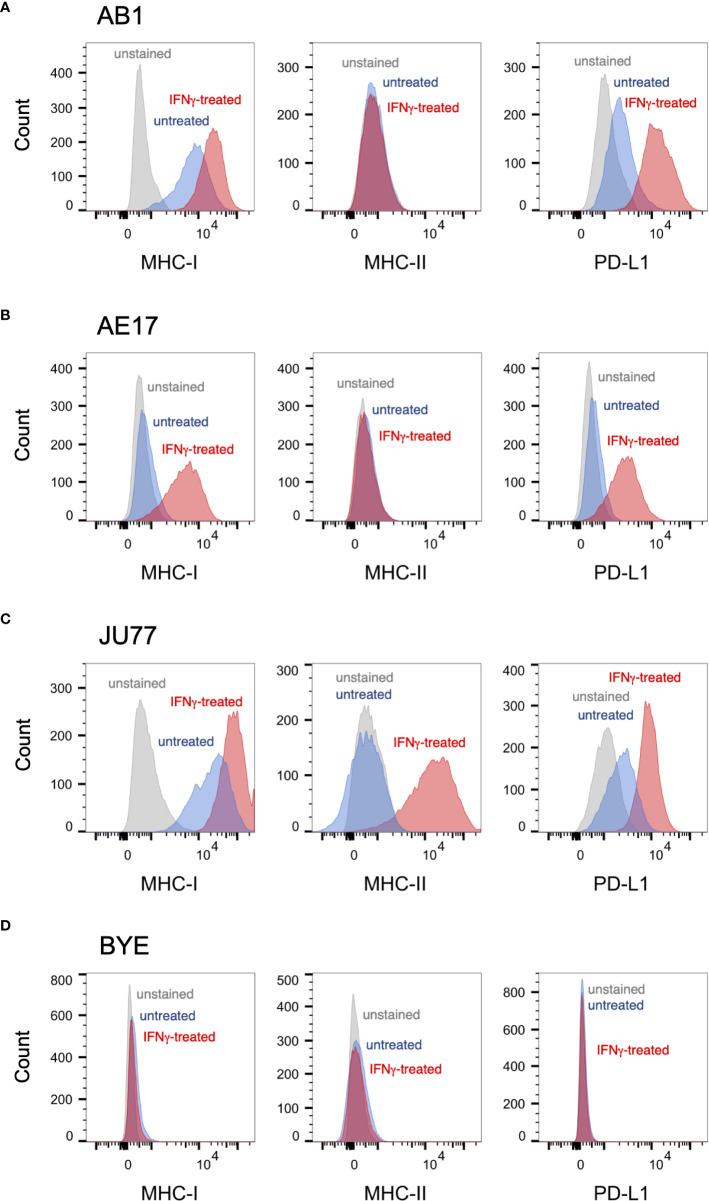
Basal expression of immune markers on mesothelioma cell lines measured by flow cytometry. **(A–D)** Representative histograms of surface MHC-I, MHC-II and PD-L1 staining on untreated mesothelioma cells (blue) against unstained controls (grey) and IFNγ-stimulated controls (red) for murine cell lines AB1 **(A)** and AE17 **(B)**, and human cell lines JU77 **(C)** and BYE **(D)**.

Prior to irradiation, we first assessed our mesothelioma cell lines for surface expression of three markers commonly found on tumor cells, and of particular relevance to cancer immunotherapy: MHC-I, MHC-II and PD-L1. These were present at varying levels of expression at baseline (**Figure 1**). In proportional terms, AB1 cells expressed high levels of MHC-I (95%) and moderate levels of PD-L1 (21%), whereas the AE17 cell line expressed negligible levels of both markers (2.6% and 1.2% respectively) (**Figures 1A, B**). Neither murine cell line expressed MHC-II on its surface (<1%). Moreover, when treated with IFN-γ, expression of MHC-I and PD-L1 was substantially upregulated on both murine cell lines, while expression of MHC-II remained unaffected. Similar to AB1, human cell line JU77 constitutively expressed high levels of MHC-I (90%) and PD-L1 (52%) but exhibited no basal expression of MHC-II (**Figure 1C**). However, when subject to IFN-γ treatment, expression of all markers was substantially upregulated on this cell line. In contrast, BYE cells showed no apparent expression of MHC-I, MHC-II or PD-L1, and IFN-γ did not induce expression of any marker (**Figure 1D**).

### Radiation leads to a dose- and time-dependent upregulation of MHC-I but not MHC-II on mesothelioma cell lines

We previously showed that effects of radiation on cell proliferation and survival saturate at doses of 8 Gy in the mesothelioma cell lines studied (26); therefore, in studying immune marker modulation cells were irradiated with a dose range of 0-8 Gy. No substantial increase in dead cells was observed 72 h after irradiation compared to unirradiated samples.

**Figure 2:**
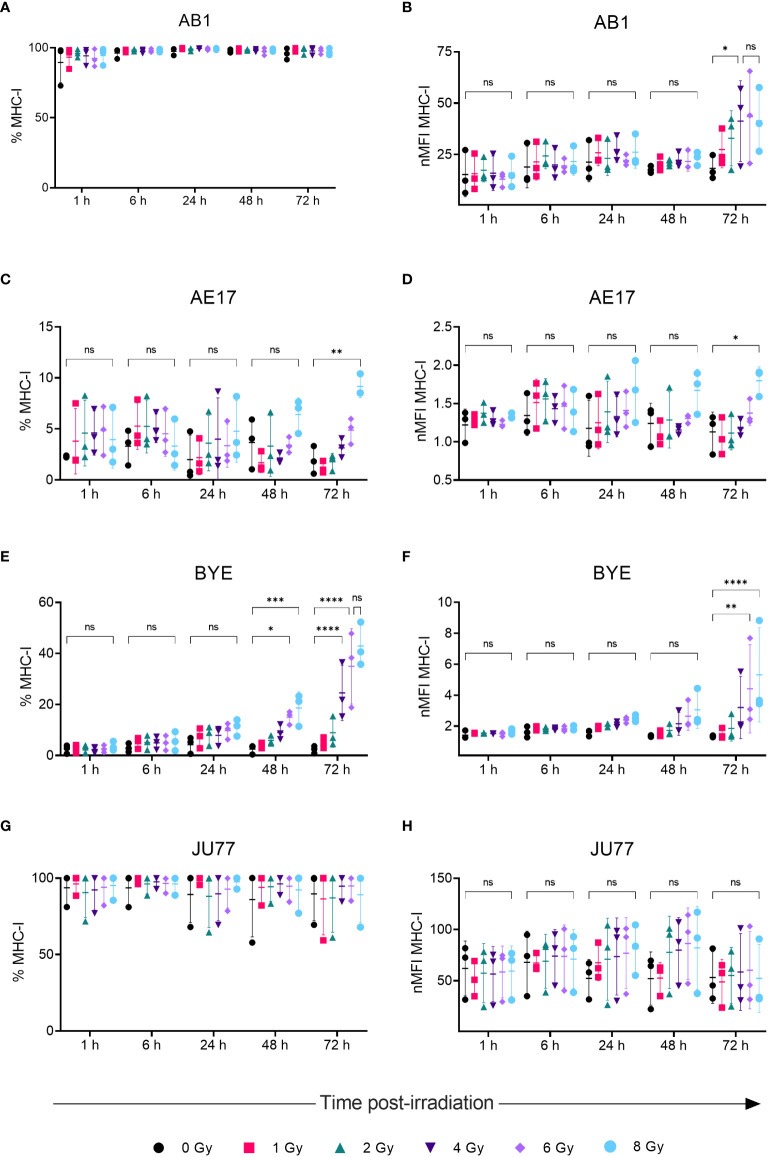
Radiation increases MHC-I expression on mesothelioma cells in a dose- and time-dependent manner. **(A–H)** Mesothelioma cells were irradiated with 0-8 Gy and changes to the percentage of MHC-I positive cells and nMFI over time (1, 6, 24, 48 and 72 h) were determined by flow cytometry for AB1 **(A, B)**, AE17 **(C, D)**, BYE **(E, F)** and JU77 **(G, H)** cell lines. Data are presented as mean ± S.D. of *n = 3* independent experimental repeats. All p-values were determined by two-way ANOVA and multiple pairwise comparison with Tukey adjustment. P-values are represented as follows: *p < 0.05, **p < 0.01, ***p < 0.001, ****p < 0.0001. ns, not significant.

A dose-dependent upregulation of surface MHC-I on both murine mesothelioma cell lines was observed following irradiation (**Figures 2A–D**). Effects were observed 72 h post-irradiation and not at earlier timepoints. A 2.5-fold increase in MHC-I MFI was found 72 h after irradiation with 4 Gy in the AB1 cell line (p = 0.0345), and this was not significantly different to expression induced by 8 Gy (**Figure 2B**). In contrast, irradiation with 8 Gy was required to induce MHC-I expression on AE17 (**Figures 2C, D**), and by 72 h was only slightly higher than unirradiated controls (1.91 ± 1.35% vs 9.15 ± 1.08%, p = 0.0015).

We also sought to compare responses to radiation in between murine and human mesothelioma cell lines. Though baseline MHC-I expression on BYE was extremely low, a stark increase in MHC-I expression was observed on this cell line following radiation (**Figures 2E, F**). Similar to murine cell lines, the effect of radiation was delayed, and observed to the greatest extent 72 h after radiation treatment. Compared to unirradiated controls, the percentage of MHC-I+ cells significantly increased 48 h after irradiation with 6 Gy (2.51 ± 1.80% vs 15.0 ± 2.67%, p = 0.019) and 8 Gy (18.6 ± 6.35%, p = 0.001). Seventy-two hours post-irradiation, however, cells irradiated with 4 Gy showed increased MHC-I (**Figure 2E**), and expression was further increased in cells irradiated with 6 Gy (35.0 ± 14.7%, p < 0.0001) and 8 Gy (42.9 ± 8.48%, p < 0.0001) (**Figure 2E**). No significant difference was found between 6 Gy and 8 Gy (**Figures 2E, F**), suggesting radiation-induced expression begins to saturate at 6 Gy. MFI levels showed similar dose- and time-related trends in expression (**Figure 2F**), with MFI increasing 4-fold after 8 Gy (p < 0.0001). In contrast, radiation did not substantially alter MHC-I expression on the JU77 cell line (**Figures 2G, H**). Also, radiation did not induce MHC-II expression on any mesothelioma cell line, regardless of dose (**Supplementary Figure 2**).

### Radiation leads to upregulation of PD-L1 on mesothelioma cell lines concurrent with MHC-I

**Figure 3:**
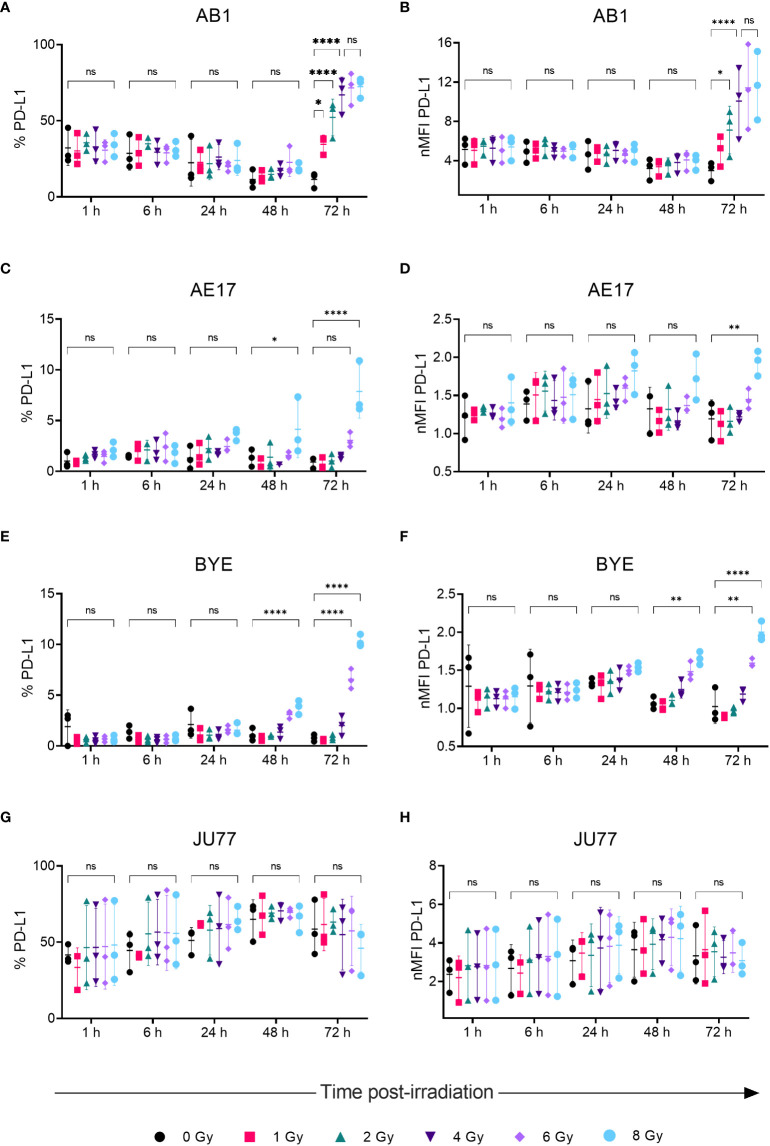
Radiation increases PD-LI expression on mesothelioma cells in a dose- and time-dependent manner. **(A–H)** Mesothelioma cells were irradiated with 0-8 Gy and changes to the percentage of PD-LI positive cells and nMFI over time (1, 6, 24, 48 and 72 h) were determined by flow cytometry for AB1 **(A, B)**, AE17 **(C, D)**, BYE **(E, F)** and JU77 **(G, H)** cell lines. Data are presented as mean ± S.D. of *n = 3* independent experimental repeats. All p-values were determined by two-way ANOVA and multiple pairwise comparison with Tukey adjustment. P-values are represented as follows: *p < 0.05, **p < 0.01, ****p < 0.0001. ns, not significant.

Radiation led to increased surface PD-L1 protein expression on AB1, AE17 and BYE cell lines in a dose- and time-related manner, concurrent with patterns in MHC-I expression. Seventy-two hours after radiation of AB1 cells both the percentage of PD-L1^+^ cells and PD-L1 MFI increased substantially (**Figures 3A, B**). This increase was apparent at lower doses (1-2 Gy) and radiation-induced upregulation saturated at 4 Gy, with five times as many cells expressing PD-L1 compared to un-irradiated controls (11.5 ± 5.00% vs 67 ± 11.7%, p < 0.0001). At the 72 h timepoint, a 3.3-fold increase in PD-L1 MFI was observed after delivery of 4 Gy, which was not significantly different to the 3.9-fold increase in PD-L1 MFI observed after 8 Gy. In contrast, 1-6 Gy did not significantly alter PD-L1 expression on AE17 cells, but expression was higher following 8 Gy radiation compared to un-irradiated samples (p < 0.05); again, this occurred only 72 h after treatment and not at earlier timepoints (**Figures 3C, D**). A similar increase in PD-L1 expression was also observed on human cell line BYE following doses of 6-Gy and above (**Figures 3E, F**). However, as with MHC-I, radiation did not significantly alter PD-L1 expression on the JU77 cell line (**Figures 2G, H**).

### Inflammatory cytokine profile following radiation differs between murine cell lines

**Figure 4:**
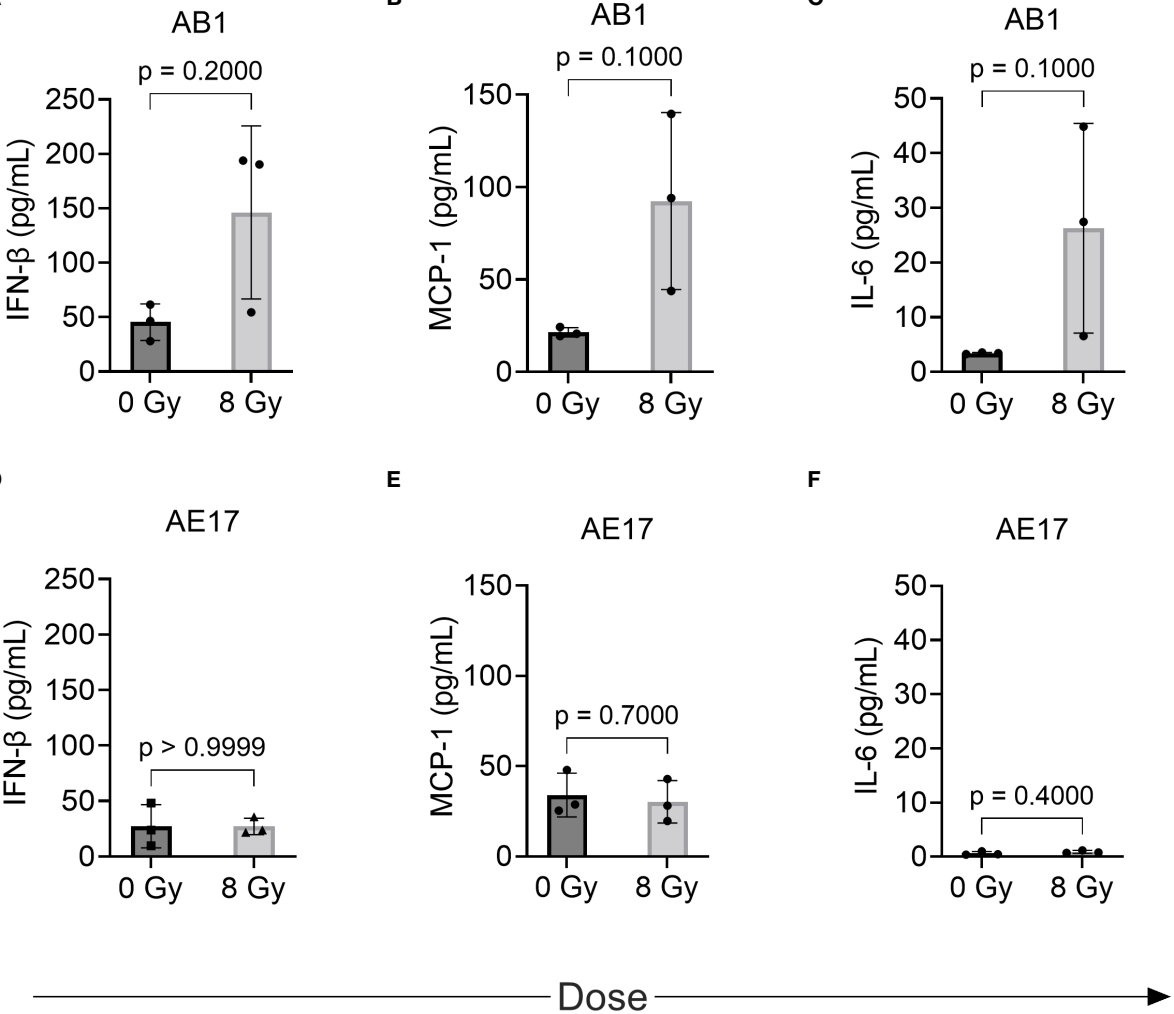
The effect of radiation on cytokine production by murine mesothelioma cell lines as measured by multiplex assay. **(A–F)** Concentrations of IFN-β, MCP-1 and IL-6 respectively in AB1 **(A–C)** and AE17 **(D–F)** cell lines, 72 h after irradiation with 8 Gy compared to sham-irradiated (0 Gy) controls. Data are presented as mean ± S.D. of *n = 3* independent experimental repeats. P-values were determined by the non-parametric Mann-Whitney U test.

We hypothesized that the radiation-induced changes in immune marker expression in murine cell lines would associate with changes in the profile of secreted inflammatory cytokines detectable in the supernatant. IL-1α, IL-1β, IL-10, IL-12p70, IL-17A, IL-23, IL-27, IFN-γ, TNF-α, and GM-CSF were not detected in the supernatant of either cell line before or after radiation treatment (**Table 1**). A non-significant increase of IFN-β, MCP-1 and IL-6 was observed following 8 Gy radiation in AB1 (**Figures 4A–C**). Interestingly, these cytokines were not upregulated in AE17 following the same dose of radiation (**Figures 4D–F**).

**Table 1:** Limit of detection **(**LOD) of undetected cytokines.

Undetected cytokines	LOD (pg/mL)
IL-23	40.40
IL-1α	0.3662
IFN-γ	3.408
TNF-α	12.60
IL-12p70	4.118
IL-1β	1.141
IL-10	19.29
IL-27	19.35
IL-17A	1.312
GM-CSF	7.643

## Discussion

ICI has transformed the landscape of cancer treatment, but sensitizing a greater proportion of patients to this therapy remains a serious challenge. RT has exhibited the potential to improve the frequency and durability of ICI responses in cancer. However, the ideal dosing and scheduling of combined RT and ICI to optimize antitumor activity, while avoiding immunosuppressive effects, remains poorly understood for many cancers including mesothelioma (27). Moreover, the radiation-induced immune-related changes at the level of tumor cells for mesothelioma have not previously been established; such information is essential to identifying potential mechanisms of synergy between RT and ICI. The present *in vitro* study therefore characterizes the dose- and time-dependent effects of radiation on expression of selected markers, crucial to immune function, in various mesothelioma cell lines with the aim of identifying an optimal radiation dose to use in future studies. Furthermore, in murine cell lines we study associated changes to inflammatory cytokine release in response to radiation, aiming to gain an understanding of the role that radiation-induced cytokines may play in the immune modulation of tumors.

Firstly, MHC-I on the surface of tumor cells is responsible for presentation of tumor-specific antigen to CD8^+^ T cells; downregulated levels of MHC-I expression typically correlate with poorer prognosis and treatment response in cancer (28). Consistent with previous studies in breast cancer (10), colon cancer (10, 11), lung cancer (10, 29) and melanoma cell lines (11), radiation increased MHC-I expression in three out of the four mesothelioma cell lines studied, presenting a potential mechanism by which radiation can sensitize tumor cells to CD8^+^ T cell-mediated killing. As expected, upregulation of MHC-I expression was dose-dependent. For the AB1 cell line, MHC-I MFI increased following 4 Gy radiation. This was not significantly different from 8 Gy and so one might conclude that a dose fraction of 4 Gy would be ideal for use in future preclinical study in this model. On the other hand, the AE17 cell line exhibited minimal basal MHC-I expression and, while 8 Gy significantly increased MHC-I expression, this occurred to a lesser extent than AB1; this may be attributed to inherent differences in radiosensitivity between mouse strains from which these cell lines were established. It is possible that higher doses may result in more pronounced marker upregulation for AE17, but as radiation is often delivered at lower doses per fraction to avoid toxicities associated with higher radiation doses, the clinical relevance and utility of this is uncertain.

Radiation-induced increases in MHC-I were accompanied by similar dose- and time-dependent changes in PD-L1 expression on AB1 and AE17 cell lines. The concurrent upregulation of the suppressive PD-L1 immune checkpoint alongside MHC-I molecules may be an adaptive mechanism to control antitumor activity, as these molecules have opposing effects on immunity. Indeed, this may indicate two different routes of immune escape resulting from selective pressure by the immune system – on the one hand MHC-I downregulation to avoid immune detection in the first place (as seen in AE17), and on the other hand constitutive elevation of PD-L1 to dampen the activity of tumor-specific T cells (as in AB1). Previous preclinical studies in other cancers including hepatocellular carcinoma (30), pancreatic ductal adenocarcinoma (16), head and neck squamous cell carcinoma (18), and non-small cell lung cancer (18, 31) have found that combining anti-PD-1 or anti-PD-L1 therapies with RT improve treatment response by circumventing this adaptive upregulation of PD-L1; such combinations may likewise be assessed in our mesothelioma models. An important factor influencing treatment response found in these studies was the ability of radiation to induce IFN-γ release by T cells to cause MHC-I and PD-L1 upregulation. Notably, IFN-γ treatment markedly increased expression of MHC-I and PD-L1 on both AB1 and AE17, underlining the importance of assessing the effect of radiation on marker expression *in vivo*, which will undoubtedly be impacted by interactions within the wider tumor microenvironment. Moreover, comparing these mesothelioma models *in vivo* will be valuable to understanding variation in responses to combined radio-immunotherapy, and the potential effects of basal and radiation-induced immune marker expression on these responses.

We also sought to compare murine mesothelioma cell lines to human cell lines. Interestingly, though MHC-I was initially poorly expressed by the BYE cell line, radiation strongly induced MHC-I expression in a dose-dependent manner. MHC-I expression was saturated at 6 Gy, showing that such effects can be achieved in humans with clinically used radiation doses. Interestingly, IFN-γ treatment did not induce MHC-I expression on BYE, suggesting an IFNγ-independent mechanism of upregulation *in vitro* for this cell line. In contrast, both MHC-I and PD-L1 were constitutively expressed by JU77 cells and could be further upregulated by IFN-γ, but expression was unchanged by any dose of radiation administered. It is possible that radiation treatment may have resulted in other immune-related phenotypic or transcriptional changes in this cell line that were beyond the scope of this study.

Optimizing the timing of delivering ICI with respect to RT is essential to improve antitumor immune responses. However, few studies have measured the dynamic changes to marker expression in response to RT; the present study therefore assessed expression at early (1-6 h) and late (24-72 h) stages post-irradiation. Importantly, we show that where modulation of immune markers is observed, this occurs maximally and, in most cases, solely at the 72 h timepoint, regardless of the level of radiation. This differs from a study in a melanoma cell line, where increased expression of cells irradiated with 10 and 25 Gy was apparent as early as 18 h after radiation; this may have been due to the higher doses administered in this study (12). Our findings may suggest the superior benefit of delivering RT prior to ICI; timing is also likely to be influenced by mechanisms of action of different ICI agents and requires further study. Whether marker expression is sustained beyond 72 h or is transient will also have implications for the relative scheduling of RT and ICI.

We hypothesized that observed increases in MHC-I and PD-L1 would associate with changes in the profile of inflammatory cytokines secreted by our cell lines. Although increased levels of MCP-1, IL-6 and IFN-β in AB1 following irradiation with 8 Gy were observed, this did not reach the set level of significance for this study. Interestingly however, the lack of change to these cytokines in AE17 corresponds with the relatively lower increases in MHC-I and PD-L1 expression observed. The increase in MCP-1 observed in AB1 is consistent with a study in a breast carcinoma cell line, where irradiation with 9 and 23 Gy significantly increased levels of this cytokine. Furthermore, IL-6 (32–35) and IFN-β (29) have previously been shown to increase tumor cell PD-L1 and MHC-I expression respectively in other cancers, and may play a similar role in mesothelioma. IL-17 (36), TNF-α (36), and IL-27 (37) can also upregulate tumor cell PD-L1 expression in various tumor cell lines, however these cytokines were not detected following irradiation of the mesothelioma cell lines in this study. It should also be noted that other cytokines that were not studied here may have the capacity to influence immune response in mesothelioma. One example is IL-15, which works to stimulate the proliferation of CD8+ T cells and natural killer cells, thereby enhancing antitumor responses (38). Administration of IL-15 superagonist and glucocorticoid-induced tumor necrosis factor receptor–related protein (GITR) agonist alongside RT improved control of irradiated AE17 mesothelioma tumor (38). The inflammatory cytokine profile and mechanisms of PD-L1 or MHC-I upregulation may also be of importance to predicting toxicities associated with delivering combined RT and ICI. A recent study of a small group of mesothelioma patients treated with radical hemithoracic RT showed that signaling pathways associated with inflammatory and fibrotic processes were upregulated in patients with no or low-grade toxicity following RT and ICI treatment, compared to those experiencing high grade toxicities (39). Overall, these results warrant further characterization of the role of these cytokines in tumor marker expression and particularly in antitumor immune responses *in vivo*.

One limitation of this study relates to our use of single-dose RT. While fractionated doses of RT are commonly used in the clinic, due to difficulties of studying fractionation *in vitro*, only single doses were administered in this study. Fractionation aims to affect a greater proportion of proliferating tumor cells by delivering multiple low doses at different times, while minimizing toxicity to healthy cells. Future work should establish whether different levels of fractionation result in more pronounced or prolonged antitumor effects compared to single doses. Nevertheless, the studied doses reflect those used in conventional fractionation (2 Gy), as well as hypo-fractionated (> 2 Gy) or hyper-fractionated (< 2 Gy) schedules.

Here, we have characterized changes in a number of crucial molecules involved in the antitumor immune response, following clinically used doses of radiation. Importantly, however, mechanisms of synergy with ICI are undoubtedly facilitated by a host of different immunomodulatory effects, such as recruitment of different immune cell subsets to tumor or remodeling of tumor vasculature, and these will require further characterization *in vivo*. Another avenue for study is the search for biomarkers to predict likelihood of response to RT and ICI, and dynamic changes to tumor or immune cell expression following radiation or ICI may be one such biomarker (27, 40). Overall, the present study lays the essential groundwork to expedite the optimization of radioimmunotherapy combinations for mesothelioma.

## Data availability statement

The raw data supporting the conclusions of this article will be made available by the authors, without undue reservation.

## Author contributions

FC contributed to study design, performed all experiments, analyzed and interpreted the data and wrote the manuscript. SK and TH assisted in preparing samples and performing experiments. AC developed the concept and design of the study, and supervised writing of the manuscript. ME, SG and AN reviewed the manuscript. All authors contributed to the article and approved the submitted version.

## Funding

This research was supported by the Cancer Australia Priority‐driven Collaborative Cancer Research Scheme (PdCCRS) (APP1163065) and the National Health and Medical Research Council (NHMRC) Centres of Research Excellence Scheme (APP1197652 and APP1107043). This material is the responsibility of the authors and does not reflect the views of these funding bodies.

## Acknowledgments

The authors acknowledge the facilities, and the scientific and technical assistance of the Centre for Microscopy, Characterization & Analysis, The University of Western Australia, a facility funded by the University, State and Commonwealth Governments. We thank staff managing the NCARD biobank for access to human cell lines in this study. We also thank Dr Tamara Abel, Dr Blake Klyen and Dr Don Heng at the Telethon Kids Institute for training and assistance with the gamma irradiator.

## Conflict of interest

The authors declare that the research was conducted in the absence of any commercial or financial relationships that could be construed as a potential conflict of interest.

## Publisher’s note

All claims expressed in this article are solely those of the authors and do not necessarily represent those of their affiliated organizations, or those of the publisher, the editors and the reviewers. Any product that may be evaluated in this article, or claim that may be made by its manufacturer, is not guaranteed or endorsed by the publisher.
