# Supplementary material for: Immune marker expression of irradiated mesothelioma cell lines
Source: Front Oncol. 2022 Oct 31;12:1020493. doi: 10.3389/fonc.2022.1020493 (PMC9659742; doi:10.3389/fonc.2022.1020493)
Supplement: Supplementary file 1 [file DataSheet_1.docx]

# Supplementary material

**Supplementary table 1.** Information on mesothelioma cell lines.

| Cell line | Source | Passage | Sex | Histology |
| --- | --- | --- | --- | --- |
| AB1 | BALB/c | 33 | F | NA |
| AE17 | C57BL/6 | 21 | M | NA |
| BYE | Patient pleural effusion | 25 | M | Epithelioid |
| JU77 | Patient pleural effusion | 73 | M | Epithelioid |

**Supplementary table 2.** Details of fluorophore-conjugated antibodies used in flow cytometry experiments.

| Fluorophore | Specificity | Reactivity | Clone | Isotype | Manufacturer | Dilution |
| --- | --- | --- | --- | --- | --- | --- |
| FITC | MHC-I | Mouse | M1/42 | Rat IgG2a, κ | Biolegend | 1/80 |
| AlexaFluor 647 | MHC-II | Mouse | M5/114.15.2 | Rat IgG2b, κ | BioLegend | 1/500 |
| PE | PD-L1 | Mouse | MIH5 | Rat SD IgG2a, λ | BD Biosciences | 1/80 |
| PE | HLA-ABC | Human | W6/32 | Mouse IgG2a, κ | BD Biosciences | 1/40 |
| PE-Cy7 | HLA-DR | Human | G46-6 | Mouse IgG2a, κ | BD Biosciences | 1/100 |
| BV421 | PD-L1 | Human |  |  | BD Biosciences | 1/100 |


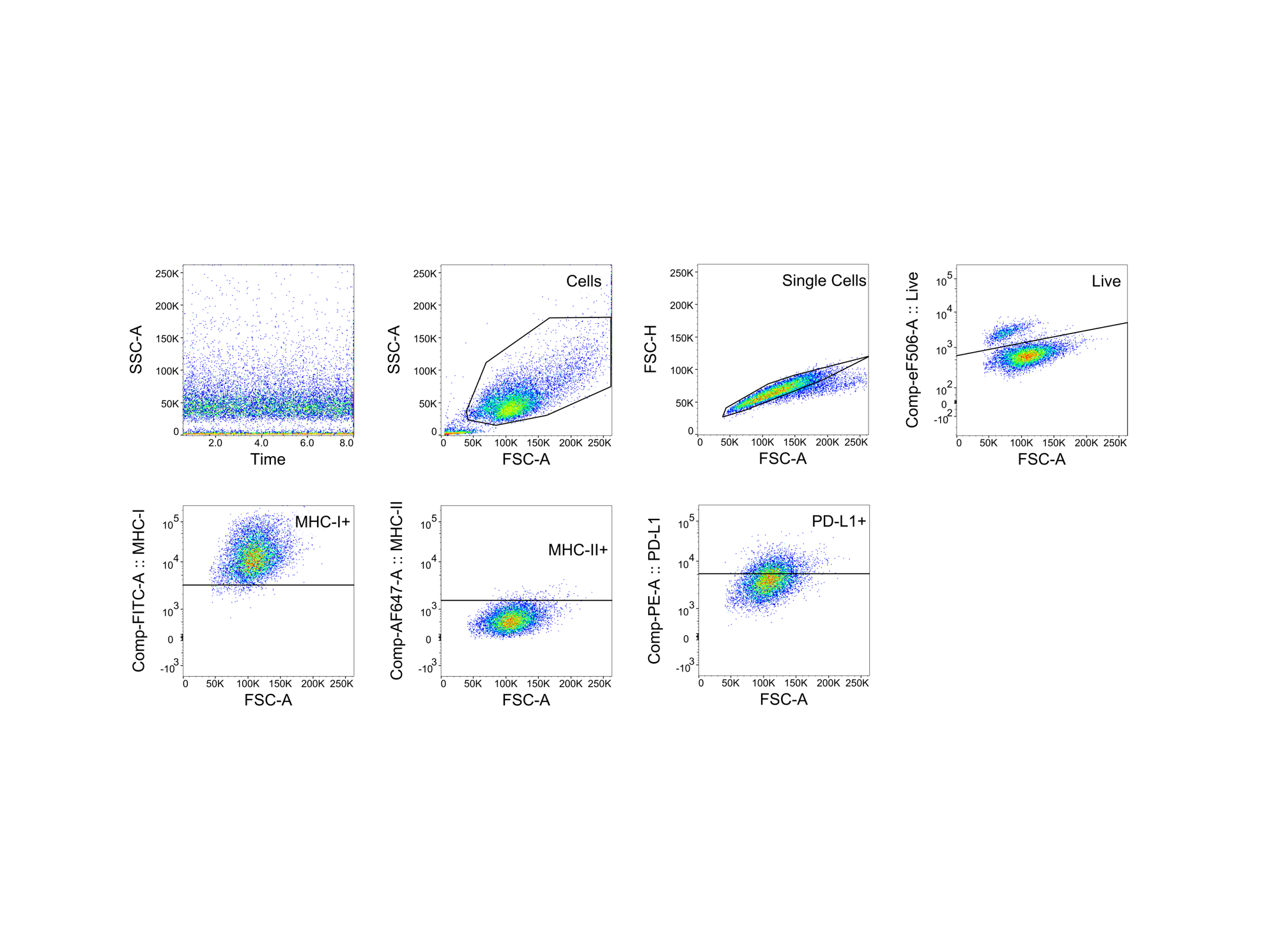


**Supplementary figure 1**. Representative flow plots showing gating strategy used for mesothelioma cell lines. A time gate was used to exclude aberrant events. Forward scatter (FSC-A) against side scatter (SSC-A) identified mesothelioma cells. FSC-A vs. FSC-H was used to exclude doublets, and viability dye eF506 was used to exclude dead cells. Gates for MHC-I, MHC-II and PD-L1 were set using fluorescence minus-one (FMO) controls.

#
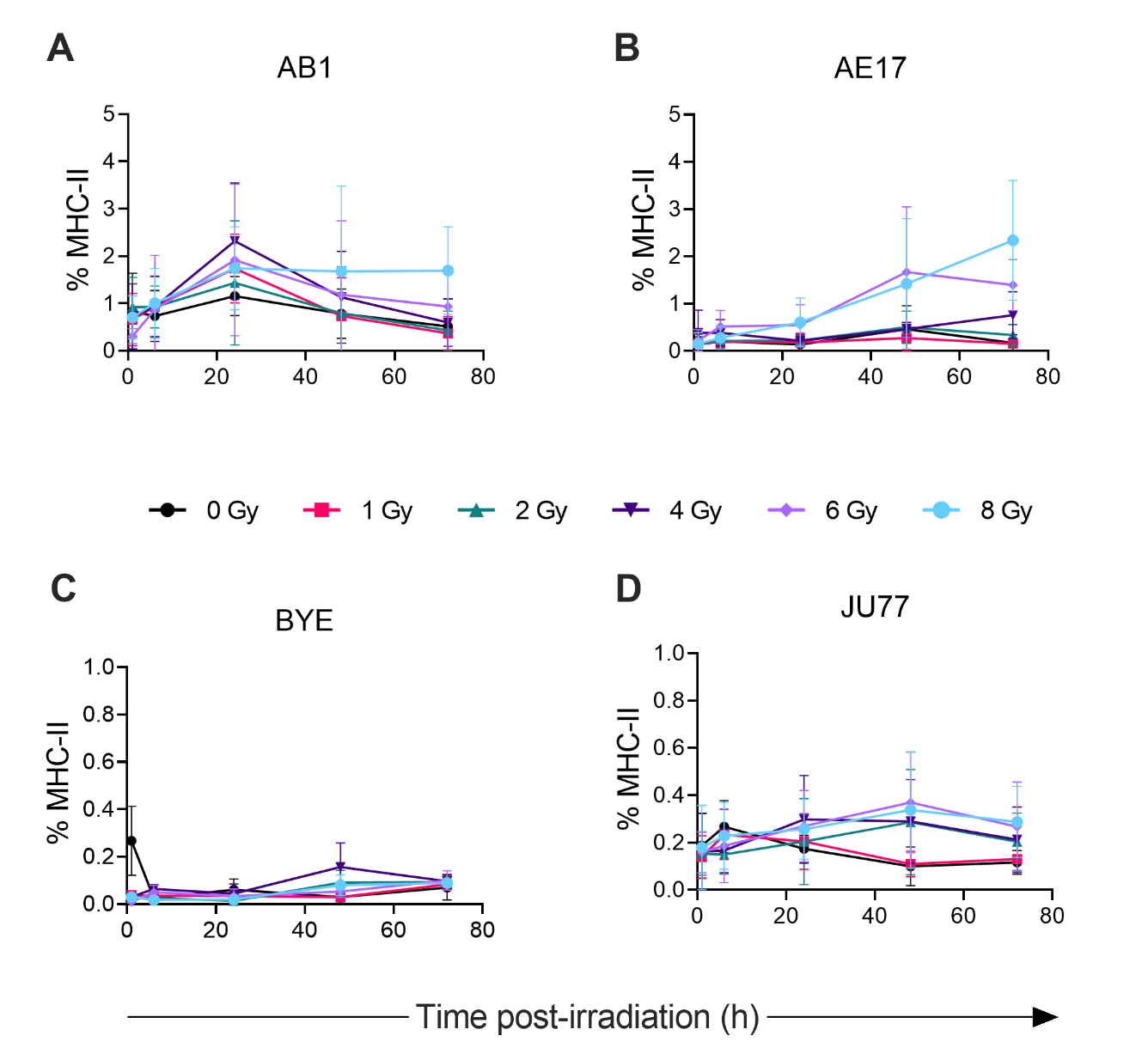


# Supplementary figure 2. Time courses showing percentage of MHC-II expression following 0-8 Gy radiation of a) AB1, b) AE17, c) BYE and d) JU77 mesothelioma cell lines, showing that radiation does not induce MHC-II expression on any cell line regardless of dose.
